# Stroke in the Setting of Giant Cell Arteritis: A Case Report

**DOI:** 10.1155/2010/549258

**Published:** 2010-12-15

**Authors:** S. McDermott, N. Casey, D. J. Robinson, K. M. Tan

**Affiliations:** Department of Medicine for the Elderly, St. Vincent's University Hospital, Elm Park, 4 Dublin 4, Ireland

## Abstract

We describe an unusual complication of a common disease: stroke presenting in a man recently diagnosed with polymyalgia rheumatica. Initial inflammatory markers were misleading. We discuss pitfalls in diagnosis, and approach to management.

## 1. Case History

A 63-year-old businessman presented to the emergency department (ED) with transient left arm weakness, expressive dysphasia, left facial droop, and muscle aches. He had attended his general practitioner (GP) one month previously with aches and pains: his GP diagnosed polymyalgia rheumatica (PMR) and commenced steroid treatment with significant improvement. Before starting steroids, erythrocyte sedimentation rate (ESR) was mildly elevated at 22 mm/hr; C-reactive protein (CRP) had been modestly elevated (46 mg/L). The patient had stopped steroids two days prior to admission, with relapse of symptoms.

His medical history was significant for ocular migraines, angina, an upper limb deep venous thrombosis 25 years ago, and a pulmonary emoblism post-angiogram 9 months ago.

On examination, reduced power was noted in his left hand with normal speech, no visual field abnormalities, and no facial droop.

**Figure 1:**
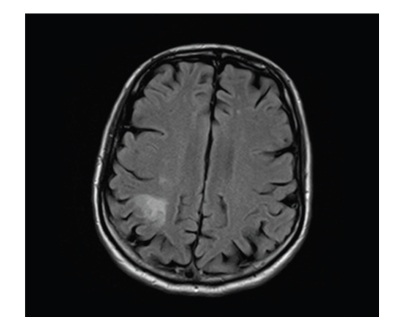
Subacute right posterior parietal infarct.

Investigations included a normal ESR and CRP. Computed tomography of brain was normal, but magnetic resonance imaging (MRI) of brain and C-spine (Figure 1) showed a subacute right posterior parietal infarct with C3-C4 posterior disc bulge. His left upper limb weakness was not attributed to cervical myelopathy. Ultrasound of carotids showed no significant stenosis. Antinuclear antibody assay was negative, as were antineutrophil cytoplasmic antibody and rheumatoid factor. A thrombophilia screen was negative.

**Figure 2:**
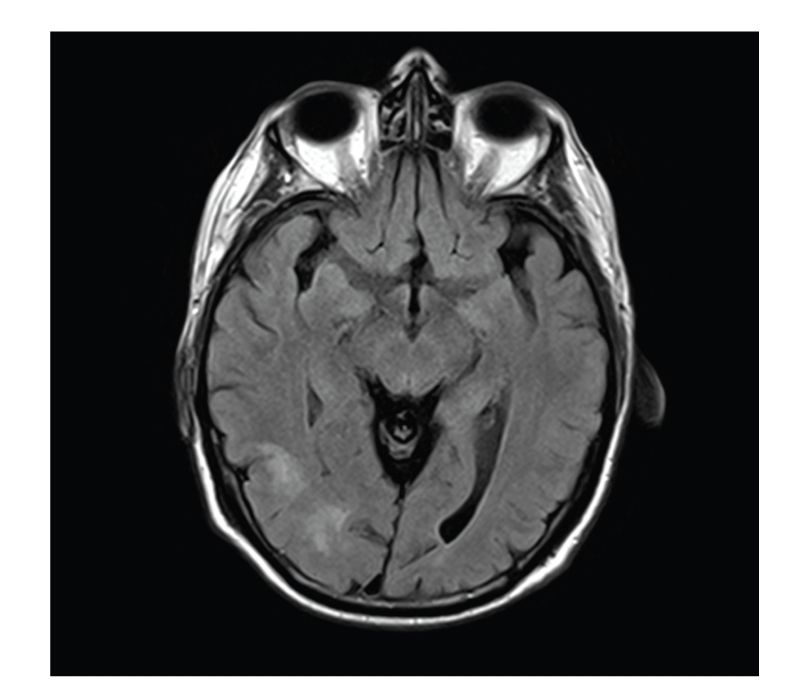
New signal abnormality in the lateral occipital lobe on the right side indicating mild interval progression of the stroke.

His symptoms were felt to be consistent with PMR and giant cell arteritis with concomitant stroke, and he was treated with high-dose aspirin and oral steroids. A temporal artery biopsy was scheduled. This was delayed secondary to worsening of left arm weakness, new left upper quadrantanopia, and left sided neglect. A repeat MRI brain (Figure 2) showed extension of the right middle cerebral infarct. CRP was raised at 41 mg/L with a normal ESR (6 mm/hr). His steroid treatment was changed to high-dose intravenous methylprednisolone 1 g daily for three days; he subsequently continued on high-dose oral steroids (prednisolone 60 mg daily). 

 A temporal artery biopsy performed two weeks post-admission disclosed a small and fibrotic vessel. Histological examination confirmed giant cell arteritis. 

 The patient was discharged with mild weakness in his left upper limb and minimal functional limitations. He continued on low-dose aspirin and oral steroids.

## 2. Discussion

PMR and giant cell arteritis (GCA) are closely related vasculitic conditions. GCA involves large-sized and medium sized arteries, most commonly the temporal arteries. PMR is characterised by aching and morning stiffness in the shoulder, pelvic girdles, and neck. The two disorders can occur separately or together and it is postulated that they are different manifestations of the same disease process [1]. About 50 percent of patients with GCA also have PMR and about 10 percent of those with PMR also have GCA [2]. 

Diagnosis is based on clinical symptoms, raised acute phase makers, response to glucocorticoids and exclusion of other disease. Different clinical criteria exist to help diagnosis: the majority (89 percent) include an elevated ESR [3]. The gold standard for diagnosis remains temporal artery biopsy.

Unusual presentations of GCA include cough, pyrexia of unknown origin, and lower limb claudication [4]. Neurological manifestations such as mononeuropathy or peripheral neuropathy can occur in approximately 30 percent of patients [5]. 

Stroke is less common (3-4 percent) [6]. Where stroke occurs, it may follow a fluctuant course corresponding to the severity of vasculitis [7]: this may have been the case with this patient. In this instance, diagnosis was complicated further by the patient's normal ESR and CRP. Low levels of ESR (<40) have been reported in up to 5.4% of patients with GCA [8]; this patient's disease seemed unusually aggressive given the low levels of antiinflammatory markers. Anticardiolipin antibodies have been associated with GCA and with more severe disease [9], however, this patient's thrombophilia screen was negative.

Patients with GCA-associated stroke tend to recover gradually with prompt administration of corticosteroids [7]. A combination of antiplatelet and corticosteroids may be advisable for preventing stroke occurrence [10].

## 3. Conclusion

Stroke is an uncommon but serious complication of GCA. Normal levels of ESR and CRP do not preclude the diagnosis. Temporal artery biopsy should be considered for patients with stroke and symptoms suggestive of PMR.

##  Conflict of Interests

None declared. The patient has given his consent for his story and his images to be used in this way.

##  Author Contribution

All authors were members of the team who treated the patient. N. Casey prepared the clinical vignette. S. McDermott conducted the literature review and prepared the first draft which was critically reviewed by D. J. Robinson and K. M. Tan. All authors approved the final draft.

## References

[1] Salvarani C, Cantini F, Hunder GG (2008). Polymyalgia rheumatica and giant-cell arteritis. *The Lancet*.

[2] Cantini F, Niccoli L, Storri L (2004). Are polymyalgia rheumatica and giant cell arteritis the same disease?. *Seminars in Arthritis and Rheumatism*.

[3] Brooks RC, McGee SR (1997). Diagnostic dilemmas in polymyalgia rheumatica. *Archives of Internal Medicine*.

[4] Unwin B, Williams CM, Gilliland W (2006). Polymyalgia rheumatica and giant cell arteritis. *American Family Physician*.

[5] Caselli RJ, Hunder GG, Whisnant JP (1988). Neurologic disease in biopsy-proven giant cell (temporal) arteritis. *Neurology*.

[6] González-Gay MA, Blanco R, Rodríguez-Valverde V (1998). Permanent visual loss and cerebrovascular accidents in giant cell arteritis: predictors and response to treatment. *Arthritis and Rheumatism*.

[7] Morris OC, Lockie P (2005). Giant cell arteritis—presenting as stroke, transient ischaemic attack and dementia. *Australian family physician*.

[8] Salvarani C, Hunder GG (2001). Giant cell arteritis with low erythrocyte sedimentation rate: frequency of occurrence in a population-based study. *Arthritis Care and Research*.

[9] Espinoza LR, Jara LJ, Silveira LH (1991). Anticardiolipin antibodies in polymyalgia rheumatica-giant cell arteritis: association with severe vascular complications. *American Journal of Medicine*.

[10] Wiszniewska M, Devuyst G, Bogousslavsky J (2007). Giant cell arteritis as a cause of first-ever stroke. *Cerebrovascular Diseases*.

